# RNA-Seq Analysis of Plant Maturity in Crested Wheatgrass (*Agropyron cristatum* L.)

**DOI:** 10.3390/genes8110291

**Published:** 2017-10-25

**Authors:** Fangqin Zeng, Bill Biligetu, Bruce Coulman, Michael P. Schellenberg, Yong-Bi Fu

**Affiliations:** 1 Department of Plant Sciences, University of Saskatchewan, 51 Campus Drive, Saskatoon, SK S7N 5A8, Canada; fangqinzeng8@gmail.com (F.Z.); bruce.coulman@usask.ca (B.C.); 2 Swift Current Research and Development Center, Agriculture and Agri-Food Canada, Box 1030, Swift Current, SK S9H 3X2, Canada; mike.schellenberg@agr.gc.ca; 3 Plant Gene Resources of Canada, Saskatoon Research and Development Centre, Agriculture and Agri-Food Canada, 107 Science Place, Saskatoon, SK S9H 3X2, Canada

**Keywords:** crested wheatgrass, *Agropyron cristatum*, late maturity, de novo assembly, differentially expressed gene, photoperiod pathway

## Abstract

Crested wheatgrass (*Agropyron cristatum* L.) breeding programs aim to develop later maturing cultivars for extending early spring grazing in Western Canada. Plant maturity is a complex genetic trait, and little is known about genes associated with late maturity in this species. An attempt was made using RNA-Seq to profile the transcriptome of crested wheatgrass maturity and to analyze differentially expressed genes (DEGs) between early and late maturing lines. Three cDNA libraries for each line were generated by sampling leaves at the stem elongation stage, spikes at the boot and anthesis stages. A total of 75,218,230 and 74,015,092 clean sequence reads were obtained for early and late maturing lines, respectively. De novo assembly of all sequence reads generated 401,587 transcripts with a mean length of 546 bp and *N50* length of 691 bp. Out of 13,133 DEGs detected, 22, 17, and eight flowering related DEGs were identified for the three stages, respectively. Twelve DEGs, including nine flowering related DEGs at the stem elongation stage were further confirmed by qRT-PCR. The analysis of homologous genes of the photoperiod pathway revealed their lower expression in the late maturing line at the stem elongation stage, suggesting that their differential expression contributed to late maturity in crested wheatgrass.

## 1. Introduction

Crested wheatgrass (*Agropyron cristatum* L.) is widely used for early spring grazing. This forage crop undergoes its initial growth in Western Canada from May to July and when it reaches the anthesis stage, its forage nutritive value and palatability declines rapidly [1]. Therefore, development of late maturing cultivars to maintain forage quality is highly desirable for this species. However, developing such cultivars is a challenging task, as plant maturity is a complex genetic trait, and little is known about genes associated with late maturity in this species [2]. 

Manipulating the timing of floral transition requires a better understanding of the molecular mechanisms of plant maturity and informative analyses of genes expressed over different stages of floral initiation and development. Flowering is one of the most important events in a plant’s life cycle [3]. In many C_3_ grasses, flowering in response to seasonal changes is controlled by pathways such as vernalization and photoperiod [4]. 

Vernalization is the prolonged exposure to the cold of a typical winter, or by an artificial equivalent to promote flowering in plants. Vernalization requirement prevents plants from flowering in the late fall before the onset of winter. In *Arabidopsis thaliana*, the activity of central flowering repressor which is encoded by the *FLOWERING LOCUS C* (*FLC*) gene was reduced by vernalization [5]. In the cereals of the grass subfamily Pooideae, particularly wheat (*Triticum aestivum* L.) and barley (*Hordeum vulgare* L.), three genes (*VERNALIZATION1* (*VRN1*), *VRN2*, and *VRN3*) have been identified and are thought to form a regulatory loop to control the timing of flowering [6]. *VRN1* encodes an AP1-related MADS-box protein [7]. *VRN2*, functioning as floral repressor FLC in *A. thaliana*, has a CCT zinc finger domain [8,9,10,11]. *VRN3* corresponds to an ortholog of *A. thaliana FT* [12]. During growth of vernalization-requiring cereals in the fall, *VRN2* represses *VRN3* to prevent flowering and *VRN1* is transcribed at very low levels [12,13,14,15]. Constitutive expression of *VRN3* from a promoter not subject to VRN2-mediated repression bypasses the vernalization requirement [12], just as the constitutive expression of *FT* bypasses FLC repression of flowering and the vernalization requirement in *A. thaliana* [16]. 

Photoperiod regulation is another major flowering time mechanism and has been studied extensively in the model plant species *A. thaliana*. The key component of the photoperiod pathway in *A. thaliana* is CONSTANS protein (CO) [16]. CO can activate the transcription of the *Flowering Locus T* (*FT*) gene. This *FT* gene is the florigen which is synthesized in leaves in long-day and transported to the shoot apical meristem to start the expression of multiple floral identity genes [17]. *CO* gene expression is regulated by light through light receptors, phytochromes (PHYs) and cryptochromes (CRYs), and by the circadian rhythm through GIGANTEA (GI) [18]. Both cryptochromes 1 and 2 (CRY1 and CRY2), which are activated by blue light, and phytochrome A (PHYA), which is activated by far-red light, stabilize the CO protein through a mechanism that disrupts the function of the CONSTITUTIVELY PHOTOMORPHOGENIC 1/SUPPRESSOR OF PHYTOCHROME A-105 complex (COP1-SPA) [19]. The circadian rhythm is an important part of the photoperiod pathway for plant flowering including *LATE ELONGATED HYPOCOTYL* (*LHY*), *CIRCADIAN CLOCK ASSOCIATED 1* (*CCA1*), *GI*, *EARLY FLOWERING 3* (*ELF3*), and *TIMING OF CAB EXPRESSION 1* (*TOC1*) [18,20,21,22,23,24,25]. The expression of *CO* is active in the same flowering-time pathway as the circadian-clock-related genes *LHY*, *GI* and *ELF3* [23,26]. Overexpression of *CO* can cause early flowering in the late flowering *lhy* and *gi-3* mutants, whereas early flowering in *elf-1* mutant correlates with elevated *CO* expression [27]. 

We initiated a research project in 2014 using the RNA-Seq technique to profile the transcriptome of flowering, analyze gene expressions for floral initiation and development with the aim to identify differentially expressed genes (DEGs) associated with late maturity in crested wheatgrass. Our research so far has generated a set of genomic resources for studying genes and pathways involved in floral transition and development, and has revealed the conserved photoperiod-circadian clock-CO-FT regulatory circuit for flowering initiation in crested wheatgrass [28]. In this communication, we will specifically report the research findings on differentially expressed genes associated with late maturity from an RNA-Seq analysis of plant maturity in early and late maturing lines. 

## 2. Materials and Methods

### 2.1. Plant Materials, RNA Extraction, and Illumina Sequencing

This RNA-Seq analysis considered two accessions: Plant Introduction (PI) No. W625134, an early maturity line, and a breeding line S9516 selected for late maturity (Table 1). The plants of these two accessions were grown in the field plots at Agriculture and Agri-Food Canada Saskatoon Research Farm, Saskatoon, SK, Canada. The field plots were established in July of 2014 with individual plants on 1 m centers. These two crested wheatgrass lines have a similar vernalization requirement to initiate flowering. The samples of the two randomly selected plants were collected at the same time, as follows: leaf tissues at stem elongation stage (vegetative stage, VS) (approximately E0), spikes at boot stage (BS) (approximately R0, 7 d after the first sampling), and spikes at the anthesis (AS) stage (R4, 30-d after the second sampling), respectively, according to Moore et al. [29]. The collected tissues were immediately frozen and stored in liquid nitrogen for RNA extraction. 

Total RNA of the early and late maturing plants was extracted from approximately 100 mg raw material at the three developmental stages, respectively, using the Qiagen RNeasy Plant mini kit (Qiagen Inc, Mississauga, ON, Canada) according to the manufacturer’s protocol. An additional DNase treatment was preformed using the Ambion DNA-free DNase treatment and removal kit (Life Technologies, Carlsbad, CA, USA) to remove additional residual genomic DNA. RNA quantification was performed using Nanodrop 8000 (Thermo Fisher Scientific, Wilmington, DE, USA). RNA 6000 Nano labchip on 2100 Agilent Bioanalyzer (Agilent Technologies, Waldbronn, Germany) was used to assess RNA integrity. The RNA samples were subsequently used in cDNA library construction. Three cDNA libraries for each line were prepared using a TruSeq^®^ RNA sample preparation Kit from Illumina (San Diego, CA, USA). Paired-end libraries were sequenced using the Illumina HiSeq^®^ 2500 system at National Research Council, Saskatoon, SK, Canada. The Sequence data were deposited in the National Center for Biotechnology Information (NCBI) Short Read Archive under the accession number SRP092070 for the early maturing line and SRP103607 for the late maturing line. 

### 2.2. De Novo Assembly, Differential Gene Expression Analysis and Sequence Annotation

De novo assembly of crested wheatgrass flowering transcriptome using six libraries of the early and late flowering plants was accomplished using Trinity with the online instructions [30,31]. Relevant transcripts were clustered into genes by Corset [32]. Bowtie2 was used to do the multi-mapping of the reads to the transcriptome and to produce the bam files for Corset analysis [33]. The de novo assembly was used as a reference for DEGs analysis. The alignment-based qualification method RSEM was used to estimate transcripts abundance [34]. Each RNA-Seq library was separately aligned to the reference, using Bowtie [35]. The DEGs were analyzed using R Bioconductor package, edgeR [36]. As there were no biological replications, the distribution of the edgeR dispersion values was empirically explored between 0.1 and 0.4. More than 10,000 DEGs were identified using edgeR dispersion value at 0.1, while 200 or fewer DEGs were found using edgeR dispersion value at 0.3. Therefore, a dispersion value of 0.2 was adopted to detect DEGs. The threshold to judge the significance of gene expression differences was “false discovery rate (FDR) ≤ 0.001 and the absolute value of Log2 fold change (Log2FC) ≥ 5.” Blast2go (http://www.blast2go.com/b2ghome) was used to align the assembled transcripts against the NCBI nr protein database for function annotation. The *e*-value cut-off was set at 1 × 10^−5^. Gene name was assigned to each gene based on top Blastx hit with the highest score. The genes related to flowering time and flowering development were explored based on the gene name.

### 2.3. The Validation of Differentially Expressed Genes 

As the original RNA-Seq analysis, done in 2014, had no biological replication, an additional effort was made here to validate a subset of the assembled gene sequences and the detected differentially expressed genes. To check the quality of assembled gene sequences, nine flowering related DEGs at the VS stage between the early and late maturing lines were selected for validation. Primer3 (http://bioinfo.ut.ee/primer3-0.4.0/primer3/) was employed for primer design, and the primers are listed in Supplementary File S1. Two early maturing plants, each from PI 439914 and PI W625134, and two late maturing plants, each from later maturing breeding lines S9580 and S9516, respectively, were randomly selected for the validation. These four plants were not genetically related to the two plants used for the RNA-Seq analysis. Total RNA at the VS stage of these four plants was extracted separately, and the extracted RNA was treated with DNAse I (Ambion Technologies, Santa Clara, CA, USA), and cDNA was synthesized using the SuperScript^®^ First-Strand Synthesis System for RT-PCR (Invitrogen, Life Technologies, CA, USA) according to the manufacturer’s instruction. The cDNA of these plants at the VS stage was employed as templates for Sanger sequencing to validate the sequences of de novo assembly. Each PCR (20 µL) contained 1× standard PCR buffer (New England Biolabs (NEB), Whitby, ON, Canada), 1 U of Taq polymerase (NEB, ON, Canada), 0.25 μM forward primer, 0.25 μM reverse primer, 100 μM each dNTP and 50 ng of genomic DNA in a total volume of 20 µL. The PCR amplification consisted of an initial denaturation at 94 °C for 3 min, 30 cycles consisting of 94 °C (30 s), 60 °C (30 s), 72 °C (2 min) terminating with 72 °C for 5 min. All PCR products were analyzed by electrophoresis in 2% agarose gels in 1× Tris-acetate-EDTA (TAE) buffer. Gels were visualized by staining in ethidium bromide and photographed on a digital gel documentation system. The DNA fragment of the expected PCR band was cloned with a pGEM-T kit (Promega, Madison, WI, USA), and the ligation product was transformed into competent *Escherichia coli* DH5α cells followed by culturing overnight at 37 °C. Positive clones containing the expected DNA fragment of DEG were identified by PCR analysis with M13 and T7 specific primers and were sequenced at the National Research Council, Saskatoon, SK, Canada. Five independent positive clones containing the DEG DNA fragment from each of the four tested plants were sequenced to ensure the right sequence obtained. The DNA sequence analysis was performed using the software Mega 6 (http://www.megasoftware.net/).

The gene expression levels of nine flowering related DEGs and three randomly selected DEGs at the VS stage between the early and late maturing lines were chosen for validation using qRT-PCR. Four plants, used for Sanger sequencing were employed for qRT-PCR validation. The sequences of the specific primer sets for each tested gene were designed using the PrimerQuest Tool (https://www.idtdna.com/Primerquest/Home/Index) and listed in Supplementary File S2. The glyceraldehyde-3-phosphate dehydrogenase (*GAPDH*) gene of crested wheatgrass (DN67262-c0-g1) was used as an internal control for normalization. Three separate first-strand cDNA reactions were analyzed in duplicate for each sample. The qRT-PCR analysis was performed with SsoFast EvaGreen supermix (Bio-Rad, ON, Canada) according to the manufacturer’s instructions using a Bio-Rad CFX96^TM^ system. KEGG Orthology Based Annotation System (KOBAS) program [37] was used for pathway enrichment analysis. Significantly enriched pathways were defined by taking a corrected *p*-value ≤ 0.05 as the threshold.

## 3. Results and Discussion

### 3.1. De Novo Assembly

The flowering date of breeding line S9516 selected for late maturity was on average 6 d later than that of the PI W625134 in the 2015 and 2016 growing seasons (Table 1). Two plants, each selected from the breeding line S9516 and PI W625134, respectively, were used for RNA-Seq analysis. Illumina sequencing generated 22,064,163; 28,810,091; and 24,343,976 clean reads for stem elongation, boot and anthesis stages of the early maturing line and 24,294,860; 27,763,034; and 21,957,198 clean reads for the late maturing line, respectively. A total of 149,233,322 clean reads for these six cDNA libraries were generated. De novo assembly of these clean reads resulted in 401,587 transcripts (Supplementary File S3) with an average length of 546 bp and *N50* length of 691 bp. The minimum and maximum lengths of the assembled transcripts were 201 bp and 15,230 bp, respectively. These transcripts were further clustered into 217,365 “genes” using Corset, with a mean length of 737 bp. Above 90% of the RNA-Seq reads of each sample mapped back to the assembly, among which more than 94% mapping was considered as properly paired reads for the assembly (Supplementary File S4). Ex*N50* value measures limit to the top most highly expressed transcripts that represent x% of the total normalized expression data and is considered to be more appropriate to evaluate the quality of the transcriptome assembly data [30,31]. In this study, about 80% of the total transcription is represented by “*E80* transcription set” of 38716 transcripts (E80N50 value of 1242 bp) (Supplementary File S5A). Based on the metric of transcripts per million (TPM), there were 94,461 “genes” with TPM > 5 and 45,689 “genes” with TPM > 10 (Supplementary File S5B). A summary of the sequencing reads, assembled transcripts and ‘genes’ for each library are presented in Table 2. 

### 3.2. Differentially Expressed Genes Detection and Annotation

**Figure 1:**
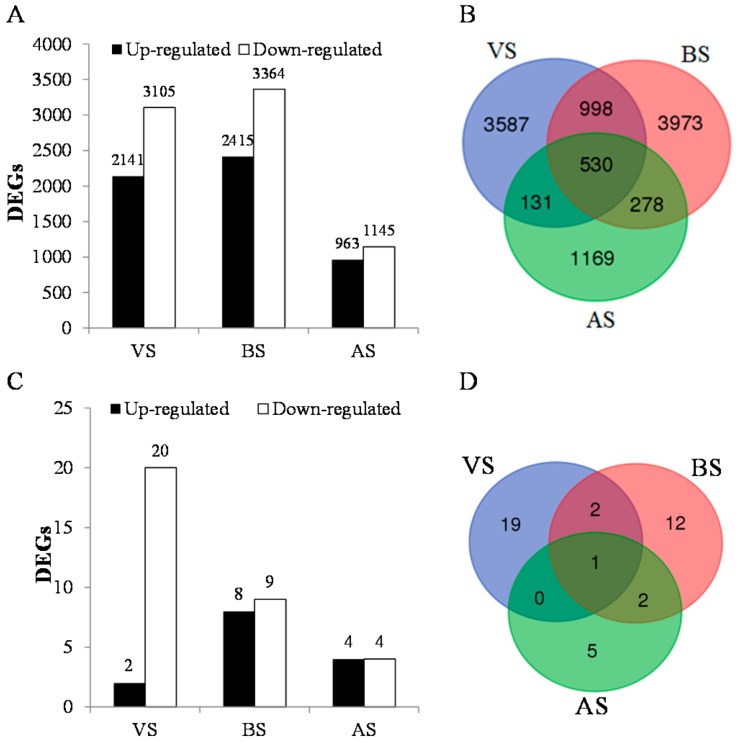
Differentially expressed genes (DEGs) between the early and late flowering lines of crested wheatgrass at three flower developmental stages: stem elongation stage (VS), boot stage (BS), and anthesis stage (AS). (**A**) Number of DEGs identified at each flower developmental stage (VS, BS, and AS) between the early and late maturing lines. (**B**) Venn diagram for the number of DEGs in three pairs of floral developmental stages (VS vs. BS, BS vs. AS and VS vs. AS). Numbers in each intersection represent the number of DEGs detected in both the stages. (**C**) Number of flowering related DEGs identified at each flower developmental stage (VS, BS, and AS) between the early and late maturing lines. (**D**) Venn diagram for the number of flowering related DEGs in three pairs of flower developmental stages (VS vs. BS, BS vs. AS and VS vs. AS). Numbers in each intersection represent the number of DEGs detected in both stages.

**Figure 2:**
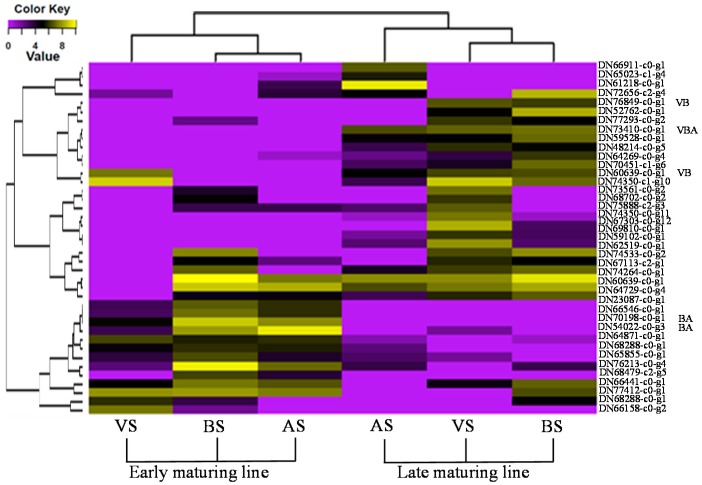
Heatmap of 40 differentially expressed genes associated with flowering between the early and late maturing lines at three developmental stages: stem elongation stage (VS), boot stage (BS), and anthesis stage (AS). Each column represents an experimental sample and each row represents a gene. Expression differences are shown in different colors. Purple means low expression and yellow means high expression. VBA: gene expressed in all three stages. VB: gene expressed at both VS and BS stages. BA: gene expressed at both BS and AS stages.

The de novo assembly of six libraries of the early and late maturing lines representing three flower developmental stages was used as reference. The expression level of each gene was calculated based on the number of reads mapping onto the reference. Comparative transcriptome profile indicated that in both genotypes, a total of 13,133 DEGs were detected at all three stages. The sequences and annotation information for these DEGs was shown in Supplementary File S6 and Supplementary File S7, respectively. Of these DEGs, 2141, 2415, and 963 were up-regulated while 3105, 3364 and 1145 were down-regulated in the late maturing line at the VS, BS and AS stage, respectively (Figure 1A; Supplementary File S8). The Venn diagram of the DEGs at three sampling stages is shown in Figure 1B. Of these DEGs, 3587, 3973, and 1169 showed specific expression at the VS, BS, and AS, respectively (Figure 1B). For each stage, the annotations and expression level fold changes of top 20 DEGs between the early and late maturing lines were listed in Supplementary File S9. These DEGs were involved in different biological pathways from disease resistance to seed maturing. To further identify biosynthetic pathways and to explore the functions of the DEGs, KOBAS program was used in the study. The DEGs at all three stages were significantly enriched to the pathways such as metabolic pathways, starch, and sucrose metabolism and photosynthesis pathways by KOBAS program (Supplementary File S10). It is worthy to note that circadian rhythm pathway was also identified by KOBAS for these up-regulated DEGs identified in the early maturing line at the VS stage (Supplementary File S10). Among these 13,133 DEGs, flowering related genes were also identified based on the function annotation. In total, 22, 17, and 8 flowering related DEGs were identified in the VS, BS, and AS stages, respectively (Figure 1C). The Venn diagram of these flowering related DEGs is shown in Figure 1D. DN73410-c0-g1 showed down-regulated expression in the late maturing line at all three stages. DN60639-c0-g1 and DN76849-c0-g1 had low expression in the late maturing line at both VS and BS stages. While DN70198-c0-g1 and DN54022-c0-g3 showed high expression in the late maturing line at both BS and AS stages (Figure 2).

### 3.3. Flowering Related Differentially Expressed Genes 

Twenty-two flowering related DEGs were identified at the VS stage and the functional annotation for these genes is listed in Table 3. DN75888-c2-g3, homologous gene to the photoreceptor *CRY1* gene which detects blue light, had around 10 times lower expression in the late maturing line. DN59102-c0-g1, homologous gene to the circadian clock gene *TIMING OF CAB* (*TOC1*), downregulated 11 times in the late maturing line. DN73561-c0-g2, which is the homologous gene of *GI*, was expressed about 10 times lower in the late flowering line. DN66104-c0-g1, DN48214-c0-g5 and DN69810-c0-g1, *COL* homologous gene, were nine times down-regulated in the late maturing line. DN67303-c0-g12, DN74350-c0-g11, and DN66158-c0-g2, homologous genes to *FT* showed around 10 times lower expression in the late maturing line. Photoreceptors, a circadian clock and an output pathway from the clock specific to flowering are composed of the photoperiod pathway [38]. The photoreceptors process physical signals and produce a circadian clock. Interactions between photoreceptors and the circadian clock are thought to allow plants to distinguish different day lengths. It has been reported in *A. thaliana* that the output from circadian clock via the GI protein activates the expression of CO [27,39]. CO then directly activates its prime target the *FT* gene which promoting flowering [35]. Overexpression of a wheat *FT* homologous gene *TaFT* in transgenic wheat caused early flowering [12]. The homologous genes of *CRY1*, *TOC1*, *GI*, *CO*, and *FT* had down-regulated expression in the late maturing line at the VS stage which indicated that differential expression of the photoperiod pathway genes could be the reason for later maturity in crested wheatgrass. Furthermore, genes involved in flower organ formation and floral development also had higher expression at the VS stage in the early maturing line. DN70348-c0-g2 and DN64729-c0-g4, the homologous genes of *MADS-box* transcription factors that were correlated with floral development, were nine times up-regulated in the early maturing line. 

Additionally, 17 and 8 flowering related DEGs were identified at the BS and AS stages between the early and late maturing lines, respectively. The function annotation of these genes is listed in Table 4 and Table 5 for BS and AS stage, respectively. DN65855-c0-g1, a *CO* homolog had about 10 times up-regulation at the BS stage and DN54022-c0-g3, homologous gene for *MADS-box transcription factor 7*, showed up-regulated expression in both the BS and AS stages in the late maturing line. 

Furthermore, the expression level changes of these 40 flowering related DEGs at all three stages between the early and late maturing lines were shown in Figure 2. The *CO* homologous gene DN69810-c0-g1 and *FT* homologous genes DN67303-c0-g12 and DN74350-c1-g11 showed specific expression at the VS stage. This is consistent with our previous report that *CO* and *FT* homologs showed specific expression in the leaves of crested wheatgrass for promoting flowering [28]. *CO* is expressed in the vasculature of *A. thaliana* leaves which can activate *FT* expression in flowering [40]. *FT* is a strong flowering promoter that is translocated from the vasculature of leaves to the shoot apical meristem [17,41]. These identified genes will be valuable for understanding the molecular basis of late maturity in crested wheatgrass.

### 3.4. Differentially Expressed Genes Validation

**Figure 3:**
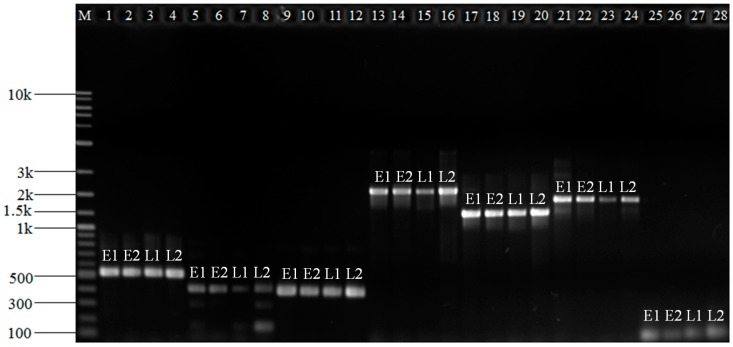
The PCR amplifications of the seven (out of nine) selected flowering-associated differentially expressed genes at the stem elongation stage of crested wheatgrass. M: DNA ladder; Lane 1–4: DN66158-c0-g2; Lane 5–8: DN67303-c0-g12; Lane 9–12: DN75888-c2-g3; Lane 13–16: DN73561-c0-g2; Lane 17–20: DN62519-c0-g1; Lane 21–24: DN66104-c0-g1; Lane 25–28: DN74350-c1-g11. E1 and E2 are two early maturing plants from PI W625134 and 439914, respectively. L1 and L2 are two late maturing plants from breeding lines, S9580 and S9516, respectively. Two primer pairs for two DEGs (DN48214-c0-g5 and DN69810-c0-g1) had no PCR amplification.

**Figure 4:**
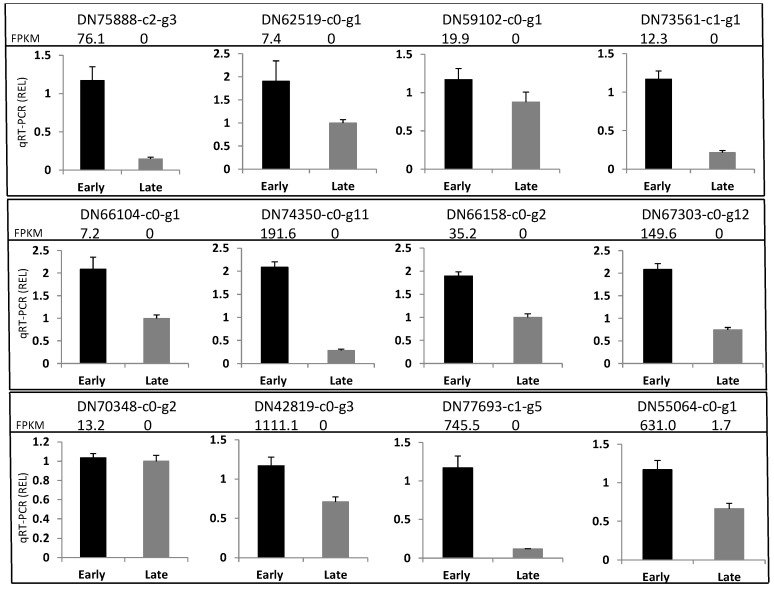
qRT-PCR analysis of the gene expression of 12 differentially expressed genes selected at the stem elongation stage between the early and late maturing lines of crested wheatgrass. Each panel shows gene ID and RNA-Seq readings of fragments per kilobase of transcript per million mapped reads (FPKM) for both lines. REL: relative expression level.

To check the quality of de novo assembly, nine flowering related DEGs at the VS stage were selected for validation. Seven of these selected DEGs had good amplification products at the expected size (Figure 3). Further Sanger sequencing indicated that they had the same sequences as that indicated by RNA-Seq de novo assembly. However, DN48214-c0-g5 and DN69810-c0-g1 had no amplification products, suggesting the presence of the poor assembled gene sequences. Twelve DEGs, nine flowering related, and three randomly selected DEGs from the up-regulated DEGs in the early maturing line at the VS stage were used to validate their expression levels. Eleven selected DEGs displayed consistent RNA-Seq results at the VS stage (Figure 4). A regression analysis of log_2_FC data from RNA-Seq and qRT-PCR revealed a trend of positive linear relationship between two measurements (Supplementary File S11). The statistical non-significance mainly reflected the test of a small number of DEGs. Together, these validation results provided valuable information on the reliability of our RNA-Seq analysis. 

### 3.5. Implications for RNA-Seq Analysis and Breeding

The research outputs presented here helped to confirm the effectiveness of RNA-Seq and differential gene expression analysis in investigation of genes related to specific traits in species without a reference genome [28]. These technologies have made the informative genomic investigation of a complex trait such as plant maturity in a non-model, polyploidy plant practically possible. However, the analysis would be more informative with proper experimental design of multiple plant pairs with extreme trait values, including biological replication [42]. 

The genomic resources generated from this RNA-Seq can be used to characterize genes associated with late maturity further in breeding materials and to develop informative markers specifically for late maturity to guide the selection of parental lines. PCR primers can be designed for the 40 differentially expressed genes associated with late maturity (Table 3, Table 4 and Table 5) and alleles associated with late maturity can be identified either using qRT-PCR or other PCR means. Informative alleles can be screened to inform parental selection for crossing. We are currently developing these single nucleotide polymorphism markers and exploring their use in our breeding program.

## 4. Conclusions

The combination of RNA-Seq application with DEG analysis based on Illumina sequencing technology has provided a comprehensive set of genomic information on gene expression level changes between the early and late maturing lines at three flowering stages. It appeared that the down-regulated expressions of the photoperiod pathway genes contributed to late maturity in crested wheatgrass. These findings not only are useful for investigating the molecular mechanism for late maturity but also can be used to develop molecular markers for selecting later maturing germplasm of crested wheatgrass.

## Figures and Tables

**Table 1:** Phenotypic characterization of the late and the early maturing lines of crested wheatgrass.

Year	Accession Name	Ploidy	Row Number	Plant Number	Heading Date ^a^	Number of Heads/Plant	Plant Height (cm)
2016	W625134 (Early)	tetraploid	18	8	26-May-2016	>20	71
2016	S9516 (Late)	tetraploid	40	30	3-June-2016	2	85
2015	W625134 (Early)	tetraploid	18	8	3-June-2015	>20	70
2015	S9516 (Late)	tetraploid	40	30	8-June-2015	11	90

^a^ Heading date: the date that a grass cultivar fully developed flower heads in spring.

**Table 2:** Summary of Illumina transcriptome sequencing from three floral development stages of crested wheatgrass: stem elongation stage (VS), boot stage (BS), and anthesis (AS) stage in the early and late maturing lines.

		W625134		S9516		
	VS	BS	AS	VS	BS	AS
Raw reads	23,073,394	29,312,848	25,603,776	25,948,120	28,249,736	22,494,862
Clean reads	22,064,163	28,810,091	24,343,976	24,294,860	27,763,034	21,957,198
Total clean nucleotides	2,727,558,541	3,563,181,662	2,966,838,914	3,005,892,104	3,432,700,188	2,714,079,492
Average read length	123.6	123.7	121.9	123.7	123.6	123.6
	All six libraries					
Total trinity transcripts	401,587					
Mean length of contigs	546					
N50 of contigs	691					
Total assembled bases	219,458,456					
“Genes” with TPM > 5 ^a^	94,461					
“Genes” with TPM > 10	45,689					

^a^ “Gene” in Trinity [30,31] means a group of transcripts clustered based on shared sequence content. TPM: transcripts per million.

**Table 3:** List of 22 (out of 5246) differentially expressed genes (DEGs) associated with flowering at the stem elongation stage (VS) between the early and late maturing lines of crested wheatgrass.

Gene ID ^a^	Putative Function	Nr ID	log_2_FC	FDR
DN67303-c0-g12	flowering locus T [*Lolium perenne*]	AIE58042.1	−11.5	1.90 × 10^−6^
DN59102-c0-g1	timing of cab expression 1	AMK48976.1	−10.9	7.50 × 10^−6^
DN74264-c0-g1	SYD isoform X1	EMT16433.1	−10.7	1.10 × 10^−5^
DN74350-c1-g11	flowering locus T [*Lolium perenne*]	AAW23034.1	−10.4	2.40 × 10^−5^
DN60639-c0-g1	transcriptional corepressor SEUSS [*Oryza sativa*]	BAJ98061.1	−10.3	2.70 × 10^−5^
DN73561-c0-g2	GIGANTEA [*Oryza sativa*]	CDM81775.1	−10.3	3.30 × 10^−5^
DN73410-c0-g1	nucleic acid binding [*Zea mays*]	BAJ87586.1	−10.0	6.50 × 10^−5^
DN75888-c2-g3	cryptochrome 2	ABX58030.1	−9.9	8.10 × 10^−5^
DN66158-c0-g2	HD3A_ORYSJ	BAH30246.1	−9.9	8.20 × 10^−5^
DN76849-c0-g1	phosphatidylinositol 4-phosphate 5-kinase 1-like	EMS61702.1	−9.7	1.23 × 10^−4^
DN62519-c0-g1	spotted leaf 11	BAJ85648.1	−9.6	1.61 × 10^−4^
DN67113-c2-g1	auxin response factor	EMT32630.1	−9.2	3.60 × 10^−4^
DN66104-c0-g1	COL10_ARATH	Q9LUA9.1	−9.2	1.12 × 10^−5^
DN68702-c0-g2	SWI SNF complex subunit SWI3B	EMT14023.1	−9.1	4.87 × 10^−4^
DN69810-c0-g1	CONSTANS CO6 [*Zea mays*]	BAJ98422.1	−9.1	4.74 × 10^−4^
DN77293-c0-g2	probable serine threonine- kinase vps15 isoform	EMT17455.1	−9.1	4.74 × 10^−4^
DN70348-c0-g2	MADS-box transcription factor 18	XP_006657934	−9.1	1.28 × 10^−5^
DN48214-c0-g5	constans-like 1 [*Picea abies*]	EMT25416.1	−9.0	5.74 × 10^−4^
DN64729-c0-g4	MADS-domain transcription factor [*Zea mays*]	ABF57916.1	−8.8	9.36 × 10^−4^
DN74533-c0-g2	phragmoplast-associated kinesin [*Oryza sativa*]	EMT21759.1	−8.8	8.80 × 10^−4^
DN66288-c0-g1	Os01g0687700 [*Oryza sativa*]	CDM83875.1	8.9	7.84 × 10^−4^
DN64871-c0-g1	gamma-glutamylcysteine synthetase	BAJ84988.1	9.3	2.75 × 10^−4^

^a^ The underlined DEGs are related to photoperiod pathway. FDR: false discovery rate.

**Table 4:** List of 17 (out of 5779) differentially expressed genes (DEGs) associated with flowering at the boot stage (BS) between the early and late maturing lines of crested wheatgrass.

			BS (Early vs. Late)	
Gene ID ^a^	Putative Function	Nr ID	log_2_FC	FDR
DN52762-c0-g1	mediator complex subunit 25	AMW92183.1	−11.4	2.59 × 10^−6^
DN59528-c0-g1	pentatricopeptide repeat-containing mitochondrial	EMT01957.1	−10.1	5.38 × 10^−5^
DN60639-c0-g1	transcriptional corepressor SEUSS [*Oryza sativa*]	BAJ98061.1	−9.6	1.90 × 10^−4^
DN64269-c0-g4	Cullin-associated NEDD8-dissociated 1	BAJ94036.1	−9.2	5.57 × 10^−4^
DN70451-c1-g6	polycomb group EMBRYONIC FLOWER 2 isoform X1	BAJ97315.1	−10.0	7.29 × 10^−5^
DN72656-c2-g4	glycerol-3-phosphate 2-*O*-acyltransferase 6	AKL71379.1	−11.5	2.02 × 10^−6^
DN73410-c0-g1	nucleic acid binding [*Zea mays*]	BAJ87586.1	−10.2	4.27 × 10^−5^
DN74350-c1-g10	flowering locus T [*Lolium perenne*]	AAW23034.1	−10.1	5.38 × 10^−5^
DN76849-c0-g1	phosphatidylinositol 4-phosphate 5-kinase 1-like	EMS61702.1	−9.3	3.78 × 10^−4^
DN65855-c0-g1	CONSTANS CO5	AAL99264.1	9.5	2.28 × 10^−4^
DN66546-c0-g1	Eukaryotic translation initiation factor 3 subunit E	EMT06719.1	10.2	5.00 × 10^−5^
DN68288-c0-g1	Peptide chain release factor 2	EMT30094.1	8.9	9.55 × 10^−4^
DN68479-c2-g5	LRR receptor-like serine threonine-kinase FEI 1	EMS63993.1	9.3	4.00 × 10^−4^
DN70198-c0-g1	WD-40 repeat-containing MSI1	ABB92268.1	11.9	9.36 × 10^−7^
DN76213-c0-g4	minichromosome maintenance 5 partial	BAJ93067.1	6.3	3.26 × 10^−5^
DN54022-c0-g3	MADS-box transcription factor 7	CAM59055.1	10.9	8.63 × 10^−6^
DN23087-c0-g1	Polyglutamine-binding 1	EMT21570.1	9.7	1.55 × 10^−4^

^a^ The underlined DEGs are related to photoperiod pathway.

**Table 5:** List of eight (out of 2108) differentially expressed genes associated with flowering at the anthesis stage (AS) between the early and late maturing lines of crested wheatgrass.

			AS (Early vs. Late)	
Gene ID	Putative Function	Nr ID	log_2_FC	FDR
DN61281-c0-g1	Aldose reductase	CDM85015.1	−7.2	7.90 × 10^−6^
DN65023-c1-g4	flower-specific gamma-thionin precursor [*Zea mays*]	BAK07823.1	−6.5	8.11 × 10^−4^
DN66911-c0-g1	flower-specific gamma-thionin precursor [*Zea mays*]	BAK07823.1	−10.4	5.20 × 10^−5^
DN73410-c0-g1	nucleic acid binding [*Zea mays*]	BAJ87586.1	−10.1	1.04 × 10^−4^
DN77412-c0-g1	SYD isoform X1	EMT16433.1	10.0	1.40 × 10^−4^
DN54022-c0-g3	MADS-box transcription factor 7	CAM59055.1	12.1	2.85 × 10^−6^
DN66441-c0-g1	flowering time control FPA isoform X1	BAJ91078.1	9.2	8.54 × 10^−4^
DN70198-c0-g1	WD-40 repeat-containing MSI1	ABB92268.1	10.3	6.58 × 10^−5^

## Supplementary Materials

The following are available online at www.mdpi.com/2073-4425/8/11/291/s1. Supplementary File S1. Primer pairs used for Sanger sequencing of nine selected DEGs; Supplementary File S2. Thirteen primer pairs used for qRT-PCR analysis of 12 selected DEGs; Supplementary File S3. Sequences of 401,587 assembled transcripts in fasta format; Supplementary File S4. Transcriptome read representation: above 90% of the RNA-Seq reads from each sample are represented by the transcriptome assembly; Supplementary File S5A. The contig N50 value is computed for cumulative sets of the most highly expressed transcripts; Supplementary File 5B. The number of transcripts that are expressed above a minimum TPM expression threshold; Supplementary File S6. Sequences of 13,133 DEGs in fasta format; Supplementary File S7. The annotation information for 13,133 DEGs in text file; Supplementary File S8. A list of genes showing differential expression between the early and late maturing lines in VS, BS, and AS, respectively, in excel file; Supplementary File S9. List the annotations and expression level fold changes of top 20 DEGs between the early and late maturing lines at each stage; Supplementary File S10. Statistically enriched pathways identified in differentially expressed genes using KOBAS database; Supplementary File S11. A regression analysis of ln(FPKM) from RNA-Seq data and REL from qPCR data.
