# Supplementary material for: RNA-Seq Analysis of Plant Maturity in Crested Wheatgrass (Agropyron cristatum L.)
Source: Genes (Basel). 2017 Oct 25;8(11):291. doi: 10.3390/genes8110291 (PMC5704204; doi:10.3390/genes8110291)
Supplement: Supplementary file 1 [file genes-08-00291-s001.zip › Supplementary Files/Supplementary_File 1.docx]

Supplementary File 1 **Primer pairs used for Sanger sequencing**

| No. | Name | Sequence(5'-3') |
| --- | --- | --- |
| 1^a^ | SNP75888F1 | CGCCTATCGAGATATCACCA |
|  | SNP75888R1 | GACCAATCGACTTCAGGAAC |
| 2^a^ | SNP74350F1 | GCCAGAACTTCAACACCAGG |
|  | SNP74350R1 | ATGCTTAATTCGTGGCTGGC |
| 3^a^ | SNP73561F1 | CATAGCCGCTGCAACTATCT |
|  | SNP73561R1 | GATGGTGTGGTGGAGTTATG |
| 4^a^ | SNP67303F1 | CCTGGTGTTGAAGTTCTGGC |
|  | SNP67303R1 | GACTCGCTGGTGGTTGGTAG |
| 5^a^ | SNP66158F1 | GCACTGGATGGTGACTGATG |
|  | SNP66158R1 | AGCTAAGTACCGTGTGATGC |
| 6^a^ | SNP62519F1 | CATATGAACGGTCGTGCATC |
|  | SNP62519R1 | GTATTGAGACGCCAGACTGT |
| 7^a^ | SNP66104F1 | GTAGTGTGCATTCGGCTAAC |
|  | SNP66104R1 | CAGATGGTGGAGAGTGATCG |
| 8^b^ | SNP69810F1 | GCGTACCGGATGGTCTTCTC |
|  | SNP69810R1 | GCCGTCCTACAGCTCCTCGT |
| 9^b^ | SNP48214F1 | GGAGGAGCACGAGGAGATGT |
|  | SNP48214R1 | AGCGGTACTGCGAACGATTG |

a: Primer pairs had amplification and confirmed by Sanger sequencing

b: Primer pairs failed to have amplification
