# Supplementary material for: RNA-Seq Analysis of Plant Maturity in Crested Wheatgrass (Agropyron cristatum L.)
Source: Genes (Basel). 2017 Oct 25;8(11):291. doi: 10.3390/genes8110291 (PMC5704204; doi:10.3390/genes8110291)
Supplement: Supplementary file 1 [file genes-08-00291-s001.zip › Supplementary Files/Supplementary_File 11.docx]

Supplementary File 11: A regression analysis of log_2_FC (fold change) data from RNA-Seq and qRT-PCR revealed a trend of positive linear relationship between two measurements.

**
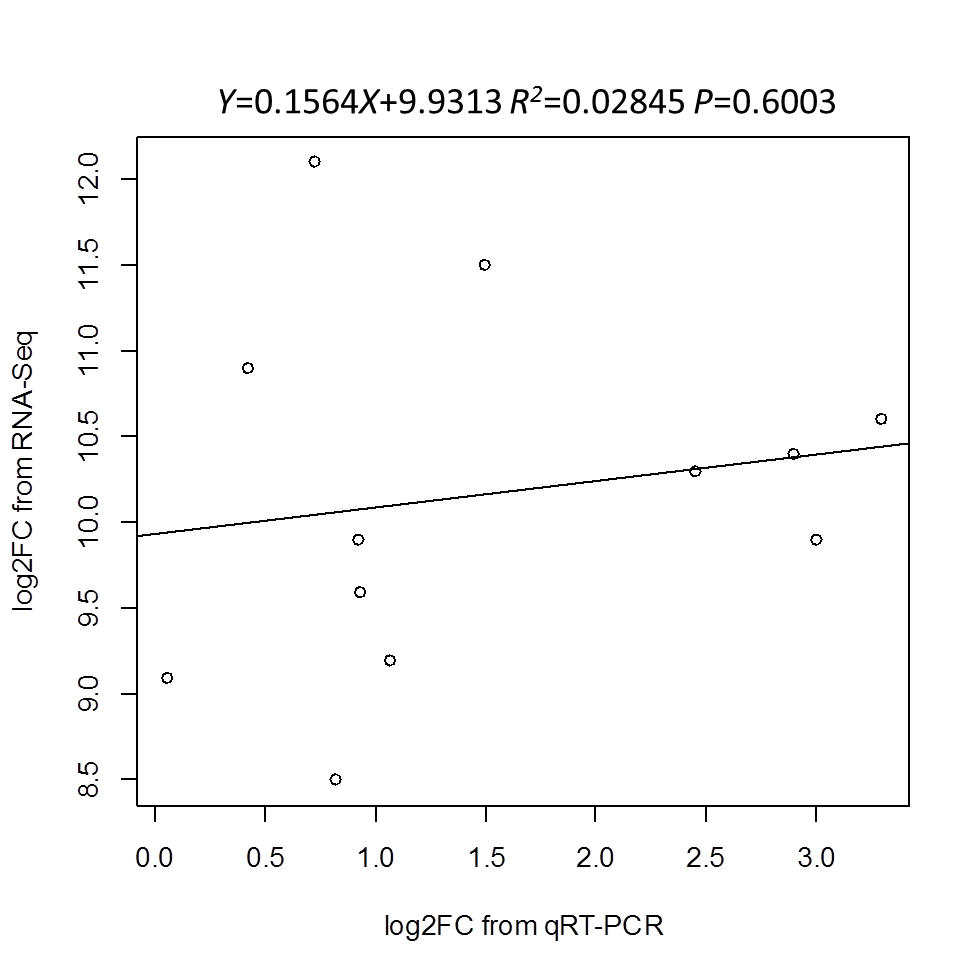
**
