# Supplementary material for: RNA-Seq Analysis of Plant Maturity in Crested Wheatgrass (Agropyron cristatum L.)
Source: Genes (Basel). 2017 Oct 25;8(11):291. doi: 10.3390/genes8110291 (PMC5704204; doi:10.3390/genes8110291)
Supplement: Supplementary file 1 [file genes-08-00291-s001.zip › Supplementary Files/Supplementary_File_2.docx]

Supplementary File 2 **Primer pairs used for qRT-PCR analysis**

| No. | Name | Sequence (5'-3') |
| --- | --- | --- |
| 1 | DN75888F2 | TTGGTGCCTGTCTCAGGTATAG |
|  | DN75888R2 | CTCAAGTGTCTTCTCCGCATTG |
| 2 | DN62519F2 | CTAGCGCAAGTGGGAGTTAAT |
|  | DN62519R2 | TCAGTCACAAGCTGGAAGAAG |
| 3 | DN59102F1 | CCACAGCAGGAGACTCATATTC |
|  | DN59102R1 | CCAATAGCACCACACTCCTT |
| 4 | DN73561F2 | GCATAAGTTGTGGGTGCTTAC |
|  | DN73561R2 | TAGATGGTGTGGTGGAGTTATG |
| 5 | DN66104F1 | TGTGACCAGCAGCTAAAGTC |
|  | DN66104R1 | CCAGAGAACGAGAGGGAAATAC |
| 6 | DN74350F2 | AGGATGTACAATTGACCTATCACC |
|  | DN74350R2 | ATTCGTGGCTGGCTTCTTAT |
| 7 | DN66158F1 | CTGCAAGGTTGATGTCTGATCTA |
|  | DN66158R1 | ACAGACATAAGTACCGGCAAAG |
| 8 | DN67303F2 | GAACACGAAGCGGTGGAT |
|  | DN67303R2 | GCACTGGCTTGTGACAGATA |
| 9 | DN70348F2 | TTGGATCCAGTACAGCTCTTTC |
|  | DN70348R2 | CTCATCGTCTTCTCCACCAAG |
| 10 | DN77693F1 | TTCTATGATCCGGCGAAGAAG |
|  | DN77693R1 | CGTCCTAGGAACTGACTGAATC |
| 11 | DN55064F1 | ACGAGCGACACGACTACTA |
|  | DN55064R1 | GAACGAGGTGGACGCATAAA |
| 12 | DN42819F1 | GGTTGAACTAAGAGGGTCATAGG |
|  | DN42819R1 | CGGTCGTTAGGAGTTGTTGT |
| 13* | GADPHF1 | GTCTGACATCGACATTGTCTCC |
|  | GADPHR1 | CAGTGGTCATCAAACCCTCAA |

*: The glyceraldehyde-3-phosphate dehydrogenase (*GAPDH*) gene of crested wheatgrass (DN67262-c0-g1) was used as an internal control for normalization.
