# Supplementary material for: RNA-Seq Analysis of Plant Maturity in Crested Wheatgrass (Agropyron cristatum L.)
Source: Genes (Basel). 2017 Oct 25;8(11):291. doi: 10.3390/genes8110291 (PMC5704204; doi:10.3390/genes8110291)
Supplement: Supplementary file 1 [file genes-08-00291-s001.zip › Supplementary Files/Supplementary_File_4.docx]

| Stage (line) | Overall alignment rate | aligned concordantly≥1 times | Proper_pairs | Improper_pairs | Left_only | right_only |
| --- | --- | --- | --- | --- | --- | --- |
|  |  |  |  |  |  |  |
| VS(Early) | 99.06% | 89.87% | 96.34% | 3.54% | 0.11% | 0.01% |
| VS(Late) | 98.76% | 83.79% | 93.98% | 5.88% | 0.12% | 0.02% |
| BS(Early) | 98.62% | 88.32% | 96.08% | 3.74% | 0.17% | 0.02% |
| BS(Late) | 98.70% | 89.88% | 96.67% | 3.15% | 0.16% | 0.02% |
| AS(Early) | 99.26% | 93.15% | 97.92% | 1.98% | 0.08% | 0.01% |
| AS(Late) | 99.09% | 87.71% | 95.96% | 3.91% | 0.12% | 0.01% |

Supplementary File 4 Transcriptome read representation: above 90% of the RNA-Seq reads from each sample are represented by the transcriptome assembly.
